# Optimizing medication appropriateness in older adults: a randomized clinical interventional trial to decrease anticholinergic burden

**DOI:** 10.1186/s13195-017-0263-9

**Published:** 2017-05-23

**Authors:** Daniela C. Moga, Erin L. Abner, Dorinda N. Rigsby, Lynne Eckmann, Mark Huffmyer, Richard R. Murphy, Beth B. Coy, Gregory A. Jicha

**Affiliations:** 1 Department of Pharmacy Practice and Science, College of Pharmacy, University of Kentucky, 789 S Limestone Street, Room 241, Lexington, KY 40536 USA; 2 Department of Epidemiology, College of Public Health, University of Kentucky, 111 Washington Avenue, Lexington, KY 40536 USA; 3 Sanders-Brown Center on Aging, 800 South Limestone Street, Lexington, KY 40536 USA; 4 PRO2RX LLC Pharmacy Consulting Services, 336 Romany Rd, Lexington, KY 40502 USA; 5 Department of Neurology, College of Medicine, University of Kentucky, 740 South Limestone Street, Lexington, KY 40536 USA

**Keywords:** Anticholinergic medication, Medication therapy management intervention, Older adults, Alzheimer’s Disease Center

## Abstract

**Background:**

The complexity of medication therapy in older adults with multiple comorbidities often leads to inappropriate prescribing. Drugs with anticholinergic properties are of particular interest because many are not recognized for this property; their use may lead to increased anticholinergic burden resulting in significant health risks, as well as negatively impacting cognition. Medication therapy management (MTM) interventions showed promise in addressing inappropriate medication use, but the effectiveness of targeted multidisciplinary team interventions addressing anticholinergic medications in older populations is yet to be determined.

**Methods:**

We conducted an 8-week, parallel-arm, randomized trial to evaluate whether a targeted patient-centered pharmacist–physician team MTM intervention (“targeted MTM intervention”) reduced the use of inappropriate anticholinergic medications in older patients enrolled in a longitudinal cohort at University of Kentucky’s Alzheimer’s Disease Center. Study outcomes included changes in the medication appropriateness index (MAI) targeting anticholinergic medications and in the anticholinergic drug scale (ADS) score from baseline to the end of study.

**Results:**

Between October 1, 2014 and September 30, 2015 we enrolled and randomized 50 participants taking at least one medication with anticholinergic properties. Of these, 35 (70%) were women, 45 (90%) were white, and 33 (66%) were cognitively intact (clinical dementia rating [CDR] = 0); mean age was 77.7 ± 6.6 years. At baseline, the mean MAI was 12.6 ± 6.3; 25 (50%) of the participants used two or more anticholinergics, and the mean ADS score was 2.8 ± 1.6. After randomization, although no statistically significant difference was noted between groups, we identified a potentially meaningful imbalance as the intervention group had more participants with intact cognition, and thus included CDR in all of the analyses. The targeted MTM intervention resulted in statistically significant CDR adjusted differences between groups with regard to improved MAI (change score of 3.6 (1.1) for the MTM group as compared with 1.0 (0.9) for the control group, *p* = 0.04) and ADS (change score of 1.0 (0.3) for the MTM group as compared with 0.2 (0.3) for the control group, *p* = 0.03).

**Conclusions:**

Our targeted MTM intervention resulted in improvement in anticholinergic medication appropriateness and reduced the use of inappropriate anticholinergic medications in older patients. Our results show promise in an area of great importance to ensure optimum outcomes for medications used in older adults.

**Trial registration:**

ClinicalTrials.gov NCT02172612. Registered 20 June 2014.

**Electronic supplementary material:**

The online version of this article (doi:10.1186/s13195-017-0263-9) contains supplementary material, which is available to authorized users.

## Background

Medication therapy is a fundamental component of the care of older patients, but evidence suggests that pharmacotherapy in this population is often inappropriate. Lau et al. [1] evaluated the magnitude of this problem in older patients followed in the National Alzheimer’s Coordinating Center cohort between 2005 and 2007. Using the Beers 2003 criteria for potentially inappropriate prescribing [2], they estimated that 20% of the older subjects without dementia and 15% of those with dementia had at least one potentially inappropriate prescription; a strong association was seen between the number of medications used and the odds of having at least one inappropriate prescription [1]. Centers for Disease Control and Prevention estimated that about 36% of adults over 60 years old in the United States used five or more drugs in the past month during 2007–2008 [3]. A recent Canadian study in frail older people found that the patients took an average of 15 medications (range 6–28), with 8.9 drug-related problems per patient (range 3–19) [4].

Different medications and medication classes are known to cause cognitive impairment that can range from acute confusion to chronic impairment. Relevant to the current study, drugs with anticholinergic properties can play an important role in causing cognitive impairment. Cholinergic antagonists deserve this special attention given the significant role played by the cholinergic system in the brain in activities such as attention, awareness, and selection of relevant stimuli in the environment [5, 6]. Anticholinergic medications have been identified as a group of drugs contraindicated in patients with dementia, largely due to their potentially severe adverse effects on cognition and psychiatric symptoms, including increasing psychotic symptoms and agitation [7, 8]. Challenge studies have shown that people with different levels of cognitive impairment are highly sensitive to anticholinergics and can experience severe central nervous system adverse effects like memory disturbances, delirium, agitation, and psychotic symptoms, including hallucinations [7]. A placebo-controlled crossover study demonstrated the significant effect of cholinergic antagonists in patients with dementia; patients enrolled in this trial experienced memory impairment, restlessness, disjointed speech, drowsiness, agitation, and hallucinations after scopolamine administration [9].

The role of medication management was formally recognized by the Medicare Prescription Drug, Improvement, and Modernization Act of 2003 that describes the role of medication therapy management (MTM) programs in reducing the risk of adverse drug events [10]. Pharmacists have been involved in different approaches for the optimization of prescribing and rational medication use in older people, and play a key role in the effective implementation of MTM programs. Previous studies brought evidence on the efficacy of collaborative, multidisciplinary (i.e., pharmacist–primary care provider) interventions to improve medication use in older patients at risk [11], and also on the importance of adopting a patient-centered approach as a fundamental philosophy of pharmaceutical care [12]. However, the literature on the impact of such interventions on reducing inappropriate anticholinergic medications and whether such interventions improve clinical outcomes (including improvement in cognitive function) is rather limited [11, 13]. Specifically, previous studies addressing MTM interventions focused primarily on polypharmacy, without specification of medication type or class, rather than the specific use of anticholinergic medication, to evaluate appropriate use [11], or have focused on patients in long-term care rather than a more generalizable population of community-dwelling older adults [13]. Effective patient-centered interventions in which a clinical pharmacist collaborates with the patient’s physician to reduce anticholinergic burden might improve cognitive performance and functionality in the older population.

Prescribing for older patients can be more challenging due to factors such as age-related changes and multiple comorbidities. The right balance between concomitantly treating several chronic conditions and avoiding medication-related negative effects is an important objective for healthcare providers, yet one that might be hard to achieve [14]. The ultimate goal of improving health outcomes in general, and brain health in particular, by optimizing medication use in older patients might be a complex process. Identifying the best approach to make this change is an essential first step en route to improving health. Therefore, we sought to investigate whether a targeted multidisciplinary team intervention would be successful at reducing inappropriate anticholinergic medication use in older patients enrolled in a cohort at the Alzheimer’s Disease Center (ADC) at University of Kentucky (UK).

## Methods

### Study design

We conducted an 8-week, parallel-arm, randomized trial to evaluate a targeted patient-centered pharmacist–physician team MTM intervention (“targeted MTM intervention”) to improve anticholinergic medication appropriateness and reduce the use of inappropriate anticholinergic medications in older patients enrolled in the ADC cohort. We defined inappropriate anticholinergic medication use in a two-step approach. All anticholinergic medications taken by each participant were labeled “potentially inappropriate” and were subject to review by the study team. The second step was conducted during the targeted MTM intervention when each of the previously flagged medications was evaluated using a risk–benefit approach with final recommendations based on the participant’s input and preference. The study was approved by the Institutional Review Board at UK. All participants provided written informed consent.

### Inclusion and exclusion

Patients were considered for inclusion in our study if they met the following eligibility criteria: actively enrolled in the ADC cohort; 65 years of age and older; reporting at least one drug with anticholinergic properties [15–17] at their annual ADC visit; and willing to participate in our intervention study. Patients were excluded if they had moderate or severe dementia as measured by a Clinical Dementia Rating (CDR) [18] global score ≥ 2, or lived in a long-term care facility at the time of enrollment.

### Participant recruitment

In short, between October 1, 2014 and September 30, 2015 we screened the records for participants actively enrolled in the ADC cohort within 1 week of their scheduled annual visit. We identified study-eligible patients who reported at least one anticholinergic drug and mailed them a letter briefly introducing our study. One to two weeks after mailing the letter, the potential participant received an enrollment call from the study team; once verbal consent for participation was granted, the medication list was finalized and additional details about the medications taken were collected. Between the enrollment call and the first study visit (2 weeks after enrollment), the pharmacist evaluated the appropriateness of the anticholinergic medication(s) and outlined points to address in case the participant was randomized to intervention.

### Randomization

After enrollment in the study, patients were randomized to either the intervention or the control group using a simple block randomization scheme (50 subjects randomized into five blocks), which was generated using the web site Randomization.com (http://www.randomization.com). The study statistician prepared 50 sealed opaque envelopes containing the sequential randomization assignments, and these were provided to the study principal investigator, who had no knowledge of the randomization assignments contained within. Each envelope was opened only after the study pharmacist completed the baseline medication review. Because the intervention was educational in nature, complete blinding of the intervention was not possible. However, we attempted to minimize potential bias and achieve the maximum level of blinding possible by this design. Specifically, when reviewing the medication list prior to the intervention, both the study pharmacist and the licensed prescriber at the ADC were unaware of the group allocation. In addition, data analysis was blinded to the intervention.

### Study procedures

**Fig. 1:**
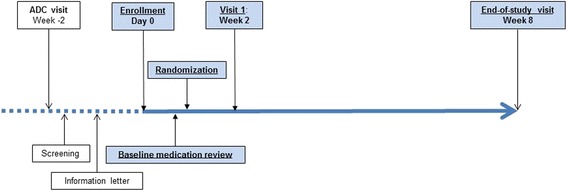
Study procedures. *ADC* Alzheimer’s Disease Center

There were two total study visits for both the intervention and control groups (Fig. 1). At the first study visit, all participants were provided with generic information available from the US Food and Drug Administration, encouraging patients to be proactive in talking to their health care providers about their medications (http://www.fda.gov/drugs/resourcesforyou/ucm079453.htm). In addition, those participants randomized to the intervention group met with the pharmacist–clinician team that conducted the targeted MTM intervention. The end-of-study visit was scheduled 8 weeks after enrollment. At this visit, all participants provided updated information on their medication use to allow us to determine any changes from baseline, and completed the Short Form Health Survey (SF-36) and the end-of-study questionnaire. Those in the control group were given the opportunity to discuss any questions or concerns about their medications with the study pharmacist.

### Intervention

The targeted MTM intervention was based on the pharmacist–clinician team drug review between enrollment and visit 1. Typical MTM interventions evaluate all medications used by a patient and determine treatment necessity and potential changes; our intervention only targeted medications known to have anticholinergic properties. For patients randomized to the intervention group, the study pharmacist provided a revised medication plan based on the drug review, which was discussed with the participant and/or their Legally Authorized Representative. Specifically, the proposed plan attempted to recommend discontinuation or replacement of any potentially inappropriate drug with anticholinergic properties, with safer drug alternatives (i.e., with less or no anticholinergic activity). When drug alternatives were unavailable, reduction in dosage was recommended whenever possible to reduce the anticholinergic burden [19]. Similar to routine clinical practice, the study clinician ultimately made the recommendations about prescription changes to the participant, while the study pharmacist was responsible for recommendations and provision of information to educate the participant about medication safety and the importance of patient involvement in medication awareness and oversight. Appropriate changes were determined by the licensed prescriber, but the participant had the freedom to accept or reject the recommendations.

### Study endpoints

The coprimary endpoints measured the impact of the targeted MTM intervention on potentially inappropriate anticholinergic use by evaluating change from baseline to end of study in: appropriateness of anticholinergic medication prescribing, as measured by the medication appropriateness index (MAI) [20]; and anticholinergic burden as measured by the number of anticholinergic drugs used and the anticholinergic drug scale (ADS) [16].

The MAI rates each medication based on 10 different criteria related to indication, effectiveness, dosing and medication-taking behavior, potential for drug–drug or drug–disease interactions, duplication of therapy, patient acceptability of the medication, and whether the medication is the least expensive option for the specific indication. Each criterion has explicit instructions and examples to guide evaluation, and the study pharmacist rates whether the particular medication was “appropriate”, “marginally appropriate”, or “inappropriate”; the final MAI score can range from 0 (totally appropriate) to 18 (completely inappropriate) [20]. MAI assessments made by a clinical pharmacist and a physician (i.e., internist and geriatrician) demonstrate high inter-rater (κ = 0.83) and intra-rater (κ = 0.92) reliability [20]. Our focus was on drugs with anticholinergic properties, either obtained by prescription or over the counter. Medication appropriateness at baseline was assessed in a blinded manner, before randomization.

Anticholinergic burden (and change from baseline to end of study) was measured using the updated version of the ADS score. The updated version of the scale was obtained from the lead author; changes from the original scale include reassigned scores for some medications (e.g., change from ADS = 0 to ADS = 1 for loratadine, or from ADS = 1 to ADS = 0 for oxazepam), and new scores for medications that were unavailable or unassessed at the time the original scale was published. The ADS has four levels for each included drug, ranging from 0 (no known anticholinergic activity) to 3 (markedly anticholinergic activity) by comparison with serum anticholinergic activity [16]. Of the existing anticholinergic scales, the ADS categorized the largest number of medications for anticholinergic activity [21]. The summation of anticholinergic activity level for all of the drugs taken by a patient reflected the total anticholinergic burden for the participant, with higher scores indicating higher burden [16].

In addition, we created a composite binary outcome measure to incorporate a change in dose when treatment discontinuation was not possible. Specifically, a reduction in anticholinergic medications (yes/no) was defined in the case of either discontinuation or dose reduction.

The secondary endpoint included the change in perceived health status from baseline to the end-of-study visit as measured using the SF-36, a validated instrument that evaluates eight health domains categorized into three major health attributes: functional status (i.e., physical functioning, social functioning, role limitations due to physical problems, role limitations due to emotional problems); well-being (mental health, vitality, bodily pain); and general health perception (an overall evaluation of health) [22]. In addition, these eight health domains can be grouped into two component scores: physical and mental health [23]. Previous research reported that the SF-36 correlated well with the Sickness Impact Profile scores, a more thorough health status evaluation [24, 25]. SF-36 summary scores range from 0 to 100, with lower scores indicating poorer perceived health. Thus, a positive change from baseline to end of study indicates improvement in perceived health. All participants completed the baseline SF-36 as part of their annual ADC assessment within the 2 weeks prior to enrollment and again at the end-of-study visit.

At the end-of-study visit, we also asked participants to complete a questionnaire asking about their experience as part of the study and how they perceived the intervention. We also investigated reasons for participation, and whether our study impacted the pattern of communication between study participants and their health care providers (i.e., physician and or pharmacist). The additional file includes the end-of-study questionnaire as used by our participants (see Additional file 1).

### Statistical analysis

We used Student’s *t* tests (or Wilcoxon rank-sum tests for nonnormally distributed variables) and chi-square or Fisher’s exact tests to assess the comparability of study groups after randomization. To examine the effect of the intervention on prescribing appropriateness measured using the MAI and ADS, we performed analysis of covariance (ANCOVA), with the dependent variables being the difference in scores between baseline and the end-of-study measure. The impact of the intervention on reducing the number of anticholinergic medications from baseline to the end of study was assessed using Poisson regression that also accounted for the number of medications the participant was taking at baseline. For our composite binary measure of reducing anticholinergic load, we conducted logistic regression; to evaluate the robustness of this measure of change, we also conducted a sensitivity analysis using logistic regression restricted to participants using moderate or strong anticholinergic medications. For the perceived health status measure, because the SF-36 does not produce a single overall measure, analysis of covariance was used to estimate the effect of the intervention on the eight SF-36 health concepts and the two component scores. For all of the analyses, our a-priori statistical analysis plan considered controlling for any variable that might have been significantly different between the two groups after randomization.

### Power calculation

Based on previous studies using the MAI as the outcome of interest, we calculated the sample size to detect a clinically relevant mean difference of 1.0 between baseline and the end-of-study assessment for the intervention group. We estimated that 34 subjects in total (17 per group) would be sufficient to detect this difference with 80% power at a significance level of 0.05. This was a rather conservative approach because previous studies showed that medication reconciliation interventions can determine a mean MAI change ranging between 1.9 and 17 [26]. In order to account for the potential loss to follow-up, we planned to enroll 25 participants for each group, for a total of 50 participants.

## Results

**Fig. 2:**
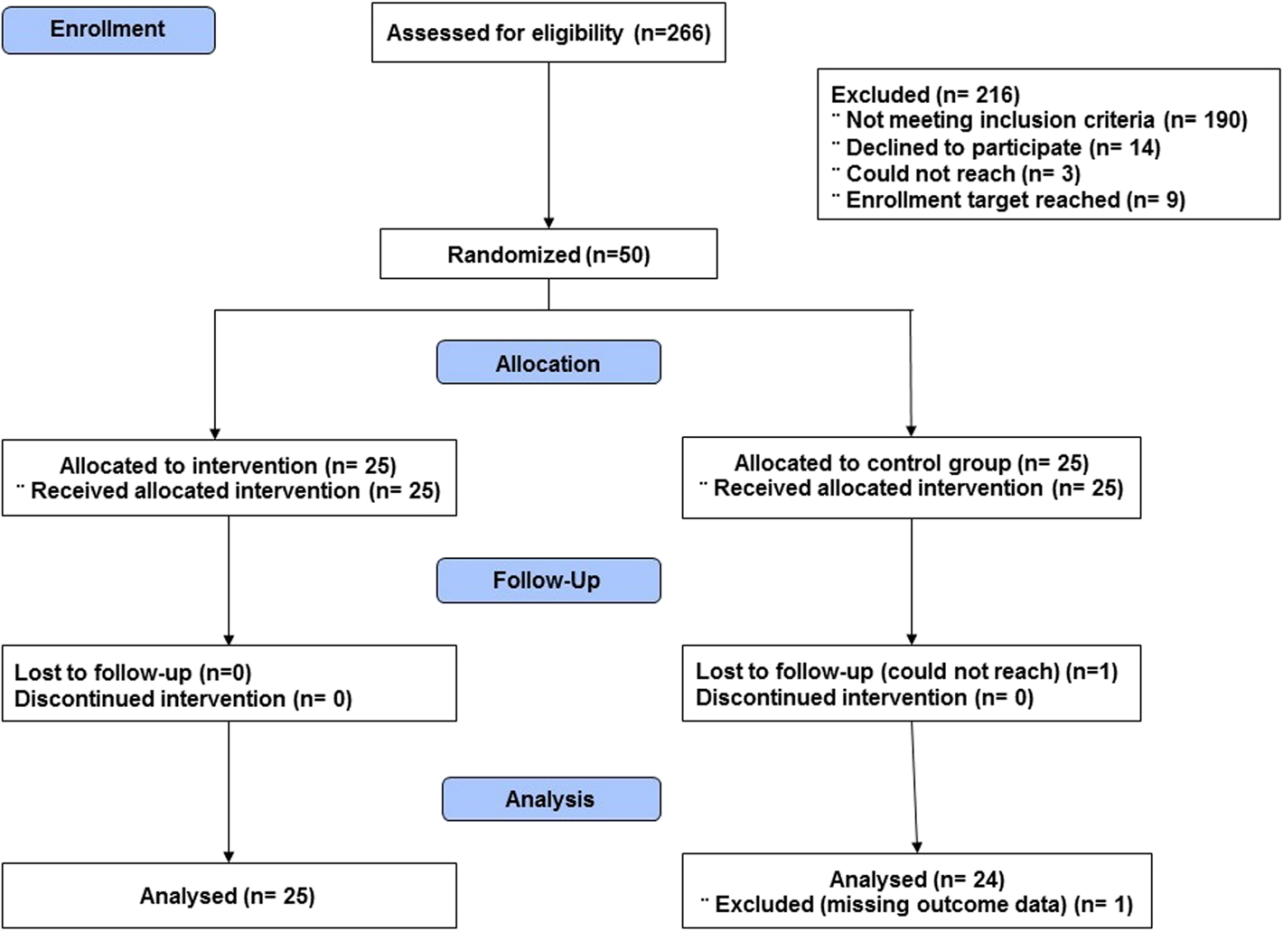
Flow diagram: study participation and selection process

Of the 266 records that were screened after a completed visit to the ADC, 50 participants were enrolled and randomized to either the control group or the intervention group (Fig. 2). One participant was lost to follow-up and was excluded from the analyses because no outcome measures were available. Of the 27 different anticholinergic medications reported at baseline and targeted for evaluation, the most frequently used by our participants were triamterene (ADS = 1, *N* = 7 (8.8%)), tolterodine (ADS = 3, *N* = 6 (7.5%)), ranitidine (ADS = 2, *N* = 6 (7.5%), and fluoxetine (ADS = 1, *N* = 6 (7.5%). Of the medications that were discontinued, eight medications were classified as ADS = 3, three were classified as ADS = 2, and six medications were classified as ADS = 1. Specific medications that were discontinued included tolterodine (*N* = 2, ADS = 3), diphenhydramine (*N* = 2, ADS = 3), fluoxetine (*N* = 2, ADS = 1), and loratadine (*N* = 2, ADS = 1).

When assessing balance between group characteristics at baseline, there were no statistically significant differences between those participants randomized to intervention and the control group (Table 1).

**Table 1:** Targeted MTM intervention: baseline participant characteristics

Demographics	All subjects (*N* = 50)	Control group (*n* = 25)	MTM group (*n* = 25)	*p* value
Age (years)	77.7 ± 6.6	79.1 ± 6.9	76.3 ± 6.2	0.13
Education (years)	16.1 ± 2.8	15.8 ± 3.0	16.5 ± 2.5	0.34
Female	35 (70.0)	17 (68.0)	18 (72.0)	0.76
Race				0.99
White	45 (90.0)	22 (88.0)	23 (92.0)	
Black or African American	5 (10.0)	3 (12.0)	2 (8.0)	
Clinical Dementia Rating				0.13
0	33 (66.0)	13 (52.0)	20 (80.0)	
0.5	12 (24.0)	8 (32.0)	4 (16.0)	
1.0	5 (10.0)	4 (16.0)	1 (4.0)	
Anticholinergic medication use				
Medication appropriateness index	12.6 ± 6.3	13.0 ± 4.4	12.2 ± 7.9	0.63
Anticholinergic drug scale	2.8 ± 1.6	2.9 ± 1.3	2.8 ± 1.9	0.73
Number of anticholinergic drugs				
1	25 (50.0)	11 (44.0)	14 (56.0)	0.40
≥2	25 (50.0)	14 (56.0)	11 (44.0)	
Used anticholinergic drugs with moderate/strong activity	31 (62)	18 (72)	13 (52)	0.14
RAND SF-36				
Health domains				
General health	67.7 ± 17.9	69.4 ± 14.8	66.0 ± 20.7	0.52
Physical functioning	67.4 ± 25.6	60.4 ± 25.9	74.2 ± 23.9	0.06
Bodily pain	66.6 ± 28.1	63.1 ± 25.8	70.0 ± 30.4	0.40
Role limitations, physical	53.1 ± 38.8	50.0 ± 33.8	56.3 ± 43.8	0.58
Mental health	82.6 ± 13.0	87.1 ± 9.4	78.2 ± 14.6	0.02
Vitality	56.1 ± 23.2	57.3 ± 23.2	55.0 ± 23.7	0.73
Social functioning	80.1 ± 22.7	80.7 ± 23.0	79.5 ± 22.8	0.85
Role limitation, emotional	81.3 ± 29.9	84.7 ± 21.9	77.8 ± 36.3	0.43
Component scores				
Physical health	63.8 ± 22.5	60.7 ± 19.1	66.8 ± 25.3	0.35
Mental health	75.0 ± 17.8	77.5 ± 13.6	72.7 ± 21.0	0.35

Data presented as mean ± SD or *N* (%)

*MTM* medication therapy management, *SF-36* Short Form Health Survey

Although CDR was not statistically significant when assessed as a three-level variable, there was a potentially meaningful imbalance such that the intervention group had more participants with intact cognition. Thus, we also assessed CDR as a dichotomous variable (CDR = 0 vs CDR > 0), and found a significant difference (*p* = 0.04). Therefore, we decided to control for CDR in all of the analyses. When evaluating the impact of the intervention on the MAI and ADS, both the unadjusted and the adjusted (controlling for baseline CDR) analyses indicated that the intervention was effective in reducing anticholinergic load (Table 2). The number of anticholinergic drugs was reduced significantly in the intervention group. The adjusted analysis controlling for baseline CDR and accounting for number of medications at baseline resulted in a rate ratio of 5.1 (95% confidence interval (CI): 1.6, 16.6), meaning the intervention group was over five times as likely as the control group to discontinue an inappropriate anticholinergic medication. Furthermore, the logistic regression model adjusted for baseline CDR indicated that the intervention group had higher odds of lowering anticholinergic load (i.e., composite outcome for change) as compared with the control group (odds ratio (OR) = 12.5; 95% CI: 2.3, 100). Similarly, when restricting the analysis to those using moderate or strong anticholinergic medications, the CDR adjusted odds of lowering anticholinergic load were higher for the intervention group (OR = 7.5, 95% CI: 1.3, 44.6).

**Table 2:** Primary outcomes: anticholinergic use change scores from baseline to end of study^a^

	Control group (*n* = 24)	MTM group (*n* = 25)	*p* value
Unadjusted outcome (mean ± SD)			
Medication appropriateness index	–1.1 ± 3.1	–4.2 ± 5.1	0.02
Anticholinergic drug scale	–0.2 ± 0.9	–1.2 ± 1.6	0.01
Number of anticholinergic drugs	–0.1 ± 0.3	–0.6 ± 0.7	0.004
Adjusted outcome^b^ (mean ± SEM)			
Medication appropriateness index	–1.0 ± 0.9	–3.6 ± 1.1	0.04
Anticholinergic drug scale	–0.2 ± 0.3	–1.0 ± 0.3	0.03
Number of anticholinergic drugs	–0.04 ± 0.03	–0.2 ± 0.1	0.04

^a^A negative result means a lower measure at the end-of-study visit (improvement in medication appropriateness index, lower anticholinergic drug scale, and fewer medications with anticholinergic properties)

^b^Adjusted for baseline Clinical Dementia Rating global score. Reduction in number of anticholinergic drugs is also adjusted for number of anticholinergic drugs taken at baseline (as the offset in the Poisson model)

When evaluating our secondary outcome of perceived health status at baseline, no significant differences were observed between the two study groups, with the exception of the mental health domain (Table 3). This difference persisted in the adjusted analysis, where the intervention group showed an adjusted mean positive change of 1.1 (SEM = 3.3), which was not statistically significant, compared with a negative change (worsening) in the control group where the mean change was –7.9 (SEM = 2.8) (*p* = 0.02). We note that adjustment for CDR changed the unadjusted point estimates for the mental health domain, but the absolute difference was similar (~11 vs ~9).

**Table 3:** Secondary outcomes: SF-36 change scores from baseline to end of study^a^

RAND SF-36	Control group (*n* = 24)	MTM group (*n* = 25)	*p* value
Health domains			
General health	–3.9 ± 14.9	4.3 ± 13.8	0.06
Physical functioning	3.6 ± 16.0	–5.2 ± 15.6	0.06
Bodily pain	1.8 ± 31.5	–6.8 ± 20.8	0.28
Role limitations, physical	4.6 ± 41.3	1.0 ± 32.5	0.75
Mental health	–6.1 ± 12.7	4.8 ± 12.5	0.005
Vitality	–4.9 ± 16.3	2.8 ± 12.9	0.08
Social functioning	–1.7 ± 29.2	0.5 ± 20.6	0.76
Role limitation, emotional	–6.1 ± 31.9	0.0 ± 32.6	0.53
Component scores			
Physical health	1.5 ± 16.5	–1.2 ± 13.2	0.53
Mental health	–4.7 ± 14.0	2.1 ± 12.9	0.09

Data presented as mean ± SD or *N* (%)

*MTM* medication therapy management, *SF-36* Short Form Health Survey

^a^A negative result means a worsening on the measure between baseline and the end-of-study visit

With regard to their reasons for participation and perceptions of the study, the majority of the participants stated either the desire to help themselves or others, or curiosity, as the main reasons for participation. Before participating in our study, 60% of the participants agreed or strongly agreed about discussing the medications they were taking with their doctor. Fewer participants agreed or strongly agreed about discussing medication-related issues with a pharmacist (32%), or with both their doctor and pharmacist (28%). After participating in the study, more participants agreed or strongly agreed that they will discuss their medication with their doctor (74%) or with their pharmacist (60%), and 52% agreed or strongly agreed that they will discuss with both their doctor and their pharmacist. Of the 50 participants enrolled in the study, 86% agreed or strongly agreed with the statement “After participating in this study I think it is important for me to understand more about the medications I am taking”, and the same proportion disagreed or strongly disagreed with the statement “Participation in this study added significant burden”.

## Discussion

We conducted a randomized intervention to evaluate the impact of a pharmacist–medical provider team impact on lowering inappropriate anticholinergic medication use in older adults. Our study showed an improvement in medication appropriateness as measured by the MAI and in anticholinergic load as measured by the number of anticholinergic medications and by the ADS. During the 8-week intervention, 44% (*n* = 11) of the intervention group stopped taking at least one anticholinergic medication, compared with just 8% (*n* = 2) of the control group. With regard to measuring quality of life, specifically the perceived health status, although only the mental health domain of the SF-36 showed a significant difference following our intervention (no change for intervention, worsening for control), it is also important to note that our targeted MTM intervention did not negatively affect our study participants.

Previous studies evaluated the impact of MTM interventions on optimizing pharmacologic therapies and reducing inappropriate medication use. For instance, Hanlon et al. [27] showed that the MAI was significantly improved following a pharmacist-led MTM intervention aimed at identifying any potential inappropriate medication used by veterans receiving care in one of the Veterans Affairs Medical Centers. Similarly, studies evaluating the effect of targeted MTM interventions on specific medications also showed promising results with significant improvement in medication appropriateness, as measured by the MAI [28, 29]. However, the evidence regarding the effect of targeted MTM interventions on reducing inappropriate anticholinergic medication use in older adults is very limited and has mainly targeted older adults in nursing homes rather than those living in the community. One study in Norway investigated whether reducing the anticholinergic burden in long-term care frail adults improves their cognitive function, and found that the intervention was successful in reducing anticholinergic load but did not show any statistically significant improvement in cognition after the 8-week follow-up [13]. A study in Finland recruited residents in assisted living facilities in Helsinki to evaluate the impact of a multidisciplinary team intervention to reduce inappropriate medication use including anticholinergic and psychotropic medications; the results published thus far are promising in that the MTM intervention resulted in a reduction of inappropriate medications [30]. Our study results are in line with the existing evidence and add important knowledge by showing that MTM interventions can be effective when targeting community-dwelling older adults.

With regard to the impact of MTM interventions on perceived health status measured using the SF-36, our results are similar to the findings from previous randomized trials that reported no statistically significant or only minimal differences for the eight SF-36 domains [27, 31–33]. In addition, a recent Agency on Healthcare Research and Quality review on MTM interventions in outpatient settings included a meta-analysis of published studies to evaluate the impact of MTM interventions on SF-36 domains and showed no statistically significant differences in all but one of the eight SF-36 domains, namely the ‘Vitality’ domain [34]. In addition, having no negative impact on any of the SF-36 domains due to the intervention is also reassuring for the potential of an MTM intervention to address inappropriate medication use.

Our study has several important strengths. The specialized care provided by clinicians at the UK ADC in collaboration with pharmacists with extensive experience in geriatric care allowed for complex medication regimens to be evaluated and modified when deemed necessary, without posing risks for participants. In addition, we were able to recruit participants in a timely fashion and had good response rates among those identified as eligible; our successful recruitment was facilitated by the collaboration with the UK ADC and the positive reputation of the center in the local community. Moreover, our loss to follow-up rate was low, with only one participant who could not be tracked for the end-of-study visit to determine outcomes. Thus, we are confident in our interpretation of the results and the fact that attrition bias was not influential.

Our study has some limitations. The 8-week follow-up did not allow us to evaluate whether the intervention is sustainable following the end of study. Participants may not be compliant with medication changes recommended by the pharmacist–clinician team in the long run, especially if primary care physicians are in disagreement with medication changes recommended by the pharmacist–clinician team. In addition, given the short follow-up, we focused on measures including the impact of the intervention on anticholinergic burden using the ADS, medication appropriateness using the MAI, and perception of health status using the SF-36. Measures of longitudinal change in objective clinical status and cognitive outcomes were not evaluated in the present study. Further research should focus on measuring appropriate objective clinical and cognitive outcomes that will necessarily involve engaging subjects over more prolonged periods of intervention in order to detect meaningful change in these important outcome measures. From a brain health perspective, our next steps would involve an expansion of the intervention to assess its impact on cognitive function. An important caveat in interpreting the present findings lies in the lack of a universally agreed-upon rating scale for anticholinergic burden. Existing anticholinergic rating scales frequently differ in the medications included and the severity of anticholinergic activity attributed to specific medications and classes of drugs. Despite this caveat, and the consideration that the scale we selected to conduct our intervention includes medications that might not be found on other anticholinergic scales [35, 36], it is important to note that the main objective of the study was to investigate whether participants were open to change medications deemed inappropriate. The fact that change was embraced for medications with low and high anticholinergic activity shows promise because it indicates willingness on the patient’s side to collaborate with a pharmacist–clinician team to maximize medication appropriateness. Lastly, the fact that our intervention was conducted by specialized providers and delivered to a highly educated and highly motivated study population may limit the generalizability of our results.

## Conclusion

Physicians regularly monitor medical treatments for their patients and change treatment plans when the balance between risks and benefits is not appropriate. However, sometimes the potential for a harmful effect is not recognized; this is often the case for some drugs that although having anticholinergic effects are not recognized as such. The inclusion of a clinical pharmacist with extensive experience in conducting medication therapy management reviews added value for the brain health care provided on a regular basis by clinicians at the UK ADC clinic. Our results bring important evidence on the feasibility of improving anticholinergic medication use in older adults living in the community, making this intervention a promising avenue for improving brain health care.

## Additional file

Additional file 1: End-of-study questionnaire. (PDF 195 kb)

## Abbreviations

ADC: Alzheimer’s Disease Center
ADS: Anticholinergic drug scale
ANCOVA: Analysis of covariance
CDR: Clinical Dementia Rating
CI: Confidence interval
MAI: Medication appropriateness index
MTM: Medication therapy management
OR: Odds ratio
SF-36: Short Form Health Survey
UK: University of Kentucky
