# Supplementary material for: Optimizing medication appropriateness in older adults: a randomized clinical interventional trial to decrease anticholinergic burden
Source: Alzheimers Res Ther. 2017 May 23;9:36. doi: 10.1186/s13195-017-0263-9 (PMC5442667; doi:10.1186/s13195-017-0263-9)
Supplement: Additional file 1: — End-of-study questionnaire. (PDF 195 kb) [file 13195_2017_263_MOESM1_ESM.pdf]

Dear study participant, we would like to learn about your experience with our study and we will really appreciate your time responding. Thank you.

1. From the list below, please rank the top three reasons why you decided to participate (1= most important 2= second most important 3= third most important).

    ____ I was curious

    ____ To help others

    ____ To help myself

    ____ I don't know

    ____ Thought it might improve my access to health care

    ____ Felt I had to

    ____ For the compensation provided

    ____ I didn't want to say no when asked to participate

    ____ Other (Please explain ________________________

The following questions deal with your reactions to participating in this study. Please circle the number that best describes your response.

	Strongly disagree (No)	Disagree	Neutral (Maybe)	Agree	Strongly agree (Yes)
2. I was glad to be asked to participate in this study.	1	2	3	4	5
3. I understood the consent form.	1	2	3	4	5
4. Before participating in this study, I used to talk to my doctor about the medications I am taking.	1	2	3	4	5
5. Before participating in this study, I used to talk to my pharmacist about the medications I am taking.	1	2	3	4	5
6. I understood the information provided by the study team.	1	2	3	4	5
7. After enrolling in this study I talked to my doctor about the medications I am taking	1	2	3	4	5
8. After participating in this study I think it is important for me to understand more about the medications I am taking.	1	2	3	4	5
9. I believe this study's results will be useful to others.	1	2	3	4	5
10. After participating in this study I will talk to my doctor about my medications.	1	2	3	4	5
11. After participating in this study I will talk to my pharmacist about my medications.	1	2	3	4	5
12. Participation in this study added significant burden.	1	2	3	4	5
13. The study procedures took too long.	1	2	3	4	5
14. Participating in this study was inconvenient for me.	1	2	3	4	5
15. I found participating in this study personally meaningful.	1	2	3	4	5
16. I gained something positive from participating.	1	2	3	4	5

17. Do you have any recommendations for us to improve this study?

____

____
